# Criteria for First-Year Growth Response to Growth Hormone Treatment in Prepubertal Children With Growth Hormone Deficiency: Do They Predict Poor Adult Height Outcome?

**DOI:** 10.3389/fendo.2019.00792

**Published:** 2019-11-26

**Authors:** Saartje Straetemans, Jean De Schepper, Muriel Thomas, Sylvie Tenoutasse, Véronique Beauloye, Raoul Rooman

**Affiliations:** ^1^Department of Pediatric Endocrinology, Maastricht University Medical Center, Maastricht, Netherlands; ^2^NUTRIM School of Nutrition and Translational Research in Metabolism, Maastricht University, Maastricht, Netherlands; ^3^The BElgian Society for PEdiatric Endocrinology and Diabetology (BESPEED), Brussels, Belgium; ^4^Department of Pediatric Endocrinology, University Hospital Brussels, Brussels, Belgium; ^5^Department of Pediatric Endocrinology, University Hospital Ghent, Ghent, Belgium; ^6^Department of Pediatric Endocrinology, Hôpital Universitaire des Enfants Reine Fabiola, Université Libre de Bruxelles, Brussels, Belgium; ^7^Unité d'Endocrinologie Pédiatrique, Cliniques Universitaires Saint-Luc, Université Catholique de Louvain, Brussels, Belgium; ^8^PendoCon, Putte, Belgium

**Keywords:** growth hormone treatment, growth hormone deficiency, children, first-year growth response criteria, adult height outcome

## Abstract

**Objective:** Several criteria for first-year growth response (FYGR) to growth hormone (GH) treatment have been proposed. We explored which FYGR criteria predicted best the final height outcome after GH treatment in prepubertal children with GH deficiency (GHD).

**Design and methods:** Height data of 129 GHD children (83 boys) who attained adult height and had been treated with GH for at least 4 consecutive years with at least 1 year before pubertal onset, were retrieved from the Belgian GH Registry. The FYGR parameters were: (1) increase in height (ΔHt) SDS, (2) height velocity (HV) SDS, (3) ΔHV (cm/year), (4) index of responsiveness (IoR) in KIGS prediction models, (5) first-year HV SDS based on the KIGS expected HV curve (HV KIGS SDS), (6) near final adult height (nFAH) prediction after first-year GH treatment. Poor final height outcome (PFHO) criteria were: (1) total ΔHt SDS <1.0, (2) nFAH SDS <−2.0, (3) nFAH minus midparental height SDS <−1.3. ROC curve analyses were performed to define the optimal cut-off for FYGR parameters to predict PFHO. Only ROC curves with an area under the curve (AUC) of more than 70% were further analyzed.

**Results:** Twelve, 22 and 10% of the children had respectively a total ΔHt SDS <1, nFAH SDS <−2, and nFAH minus midparental height SDS <−1.3. The AUC's ranged between 73 and 85%. The highest AUC was found for first-year ΔHt SDS to predict total ΔHt SDS <1, and predicted nFAH SDS to predict nFAH SDS <−2. The currently used FYGR criteria had low specificities and sensitivities to detect PFHO. To obtain a 95% specificity, the cut-off value (and sensitivity) of FYGR parameters were: ΔHt SDS <0.35 (40%), HV SDS <−0.85 (43%), ΔHV <1.3 cm/year (36%), IoR <−1.57 (17%), HV KIGS SDS <−0.83 (40%) to predict total ΔHt SDS <1; predicted nFAH SDS (with GH peak) <−1.94 (25%), predicted nFAH SDS (without GH peak) <−2.02 (25%) to predict nFAH SDS <−2. At these cut-offs, the amount of correctly diagnosed poor final responders equals the amount of false positives.

**Conclusion:** First-year growth response criteria perform poorly as predictors of poor final height outcome after long-term GH treatment in prepubertal GHD children.

## Introduction

Growth hormone deficiency (GHD) in children is mostly idiopathic and is treated with daily growth hormone (GH) injections for a mean duration of 4 to 11 years (1–8). GH treatment is therefore not only burdensome for the patients and their families, it is also costly. In addition, not every child benefits from GH treatment and the poor responder rate in GHD has been found to be between 10 and 30% (9, 10). It is therefore common practice to evaluate the response to GH therapy after 1 year to detect poor responders in order to reassess the diagnosis, adapt the GH dose or stop the treatment to avoid unnecessary daily injections and expenses. The evaluation is usually done after 1 year of treatment because it is known that the first year response is an important determinant of the total treatment height outcome (11).

Several methods exist to evaluate this first year response such as increase in height (ΔHt) SDS, Δ height velocity (HV), HV SDS on the population HV reference curve, and HV SDS on the predicted HV for idiopathic GHD curve (12, 13). A parameter (index of responsiveness, IoR) has been introduced that compares the observed first year HV to a predicted HV derived from prediction models (14, 15). More recently, models have been proposed that predict the near final height outcome after the first treatment year (16). All these methods for evaluation of first-year growth response use arbitrary decision values that are not based on their ability to predict a final height outcome. Up to now, the value of these first-year growth response and responsiveness parameters as predictors of a poor final height outcome after long-term GH treatment in GHD patients has not been analyzed.

We therefore set out to determine the sensitivity and specificity of these first year growth response (FYGR) criteria at their proposed threshold levels to detect a poor final height outcome (PFHO), defined by different criteria. In addition, we performed ROC analyses to calculate the decision levels at a desired 95% specificity.

## Materials and Methods

### Materials

The auxological data and GH treatment characteristics of prepubertal children diagnosed with GHD, who were enrolled in the Registry of the BElgian Society for PEdiatric Endocrinology and Diabetology (BESPEED) since 1986, were retrieved. This registry was approved by the ethical committee of the Brussels University and the University Hospital Brussels in Belgium. The legal representatives of all subjects gave written informed consent to have their data registered in a national registry and to use their data for scientific purposes in accordance with the Declaration of Helsinki. All data are pseudonymised to comply with rigorous privacy guidelines. Only patients, who had been treated with recombinant human GH on a daily regimen for at least 4 consecutive years and at least 1 year before pubertal onset and who had attained final adult height were included. Growth hormone was only of recombinant origin in all cases. GHD patients with and without developmental anatomical anomalies of the pituitary were included, but those with acquired GHD were excluded. Other exclusion criteria were any medication or medical condition other than GHD that can affect growth, interruption of GH treatment for more than 6 months, and smallness for gestational age. In total, 129 patients (83 males and 46 females) with GHD (81 with isolated GHD and 48 with multiple pituitary hormone deficiency) met the inclusion and exclusion criteria.

### Methods

The diagnosis of GHD was made by the treating physician and peer-reviewed at the monthly meeting of BESPEED, according to the KIGS etiology classification system (17). All patients had a peak GH concentration of < 10 μg/l after glucagon and/or insulin stimulation. Pubertal onset was defined as testicular volumes ≥ 4 ml for boys and Tanner breast stage ≥ 2 in girls.

Variables retrieved from the registry were (a) status at birth: sex, birth weight and length; (b) father's and mother's height (Ht); (c) pre-treatment Ht when measured between 6 and 18 months before GH treatment; (d) patient variables at the start of the treatment period: chronological age, Ht, weight (Wt), body mass index (BMI), the highest peak GH concentration during a provocation test, the presence of other pituitary hormone deficiencies, and (e) treatment modality: average GH dose (μg/kg.day) during the first year of GH treatment.

Birth weight for gestational age was transformed into SDS, based on the standards of Niklasson et al. (18). Midparental height (MPH) was calculated as follows: (father's Ht + mother's Ht + 13 for boys/−13 for girls)/2 (19). Height, weight, BMI, MPH, and HV were converted to SDS using Flemish reference data by Roelants et al. (20).

Near (n) FAH was defined as the height attained when HV was less than 2 cm/year, calculated over a period of minimum 9 months, and when the child had a chronological age >17 years in boys and >15 years in girls. nFAH SDS was calculated in 2 different ways: (1) using the chronological age (CA), (2) using the growth reference data at age 21 years (A21).

The FYGR parameters were: (1) increase in height (ΔHt) SDS, (2) height velocity (HV) (cm/year), (3) HV SDS, (4) ΔHV (cm/year), (5) index of responsiveness (IoR) in KIGS prediction models, (6) first-year HV SDS based on the KIGS expected HV curve (HV KIGS SDS), (7) near final adult height (nFAH) prediction after first-year GH treatment.

First-year gain in height (ΔHt) SDS and first-year HV (cm/year), were calculated as the increment in height between start and after minimum 9 months and maximum 15 months of GH therapy and subsequently scaled to 12 months. ΔHV (cm/year) was calculated as the HV during the first year of GH treatment minus the HV during the pretreatment year. The HV during the first year of GH treatment was plotted on the Flemish HV curve (20), and on the reference curve for the HV during the first year of GH treatment developed by Ranke et al. (15), and its SDS value was calculated. Predicted HV was calculated using the KIGS prediction models for idiopathic GHD (14, 15), if all parameters required for the mathematical algorithm were available. Differences between observed and predicted HVs were expressed as index of responsiveness (IoR), calculated as the observed HV minus the predicted HV, divided by the SD of the predicted HV of the child. The predicted nFAH was calculated after the first year of GH treatment, using the prediction models by Ranke et al. (16). For the prediction models, observed heights (height at start, height after first year, parental heights, and nFAH) were converted to SDS using reference data by Prader et al. (21) and the MPH SDS was calculated with the Cole formula: (father height SDS + mother height SDS)/1.61.

The long-term growth response to GH was evaluated by three different, but complementary methods: (1) nFAH, expressed as a height SDS; (2) total ΔHt SDS, calculated as the nFAH SDS minus height SDS at start of GH treatment; (3) nFAH SDS minus MPH SDS, an index of achieving genetic height potential.

A poor near final height outcome to GH treatment was defined as: (1) total ΔHt SDS < 1, (2) nFAH <−2 SD of the population mean, or (3) nFAH SDS minus MPH SDS <−1.3.

### Statistical Analysis

The variables are reported as the median (25–75th percentile) and mean (±SD). A Shapiro-Wilk test was used to test for the normal distribution. Differences between groups were tested with a *t*-test when the distribution of data was normal, and with a Mann-Whitney *U*-test otherwise. ROC curve analyses were performed to examine the relationship between sensitivity and specificity for the different FYGR parameters and PFHO criteria and to determine the test cut-off values that had a 95% specificity. The minimum AUC was set at 0.7. Significance was considered at the 5% level (*p* < 0.05). MedCalc® and IBM SPSS Statistics 25® software was used for all statistical analyses.

## Results

### Background Characteristics

The background and auxological characteristics of 129 included GHD children (83 males, 46 females) are listed in Table 1. GH therapy was initiated at a mean age of 6.8 years, a median height SDS of −3.31 and a median height minus MPH SDS of −2.34. The mean GH dose at start was 28 μg/kg.day.

**Table 1:** Characteristics: background, at GH start, after first year, at nFAH.

	**n**	**Median**	**p25**	**p75**	**Mean**	**SD**
**Background**						
Gestational age, weeks	123	40.0	38.0	40.0	38.7	2.8
Birth weight, SDS	122	−0.29	−0.86	0.34	−0.20	0.89
Birth length, SDS	110	−0.38	−1.02	0.46	−0.25	0.96
Father height, SDS	124	−1.20	−1.79	−0.19	−1.03	1.17
Mother height, SDS	124	−0.78	−1.62	−0.27	−0.91	1.13
MPH, SDS	124	−1.10	−1.70	−0.41	−0.99	0.93
maximum GH peak, μg/l	129	4.0	2.1	6.9	4.4	2.7
**At start GH treatment**						
Age, years	129	6.6	4.7	8.7	6.8	2.6
Height, SDS	129	−3.31	−3.89	−2.73	−3.39	0.85
Height minus MPH, SDS	124	−2.34	−2.99	−1.71	−2.39	1.07
BMI, SDS	129	−0.42	−1.20	0.34	−0.36	1.11
GH dose, μg/kg.day	129	27.0	24.5	31.1	28.0	5.4
HV during pretreatment year, cm/year	107	5.0	3.8	6.0	5.2	2.0
**After first-year GH treatment**						
Height, SDS	129	−2.34	−2.80	−1.90	−2.39	0.80
Height minus MPH, SDS	124	−1.29	−1.98	−0.74	−1.38	0.94
Δ BMI, SDS^a^	129	−0.21	−0.56	0.07	−0.27	0.57
*Growth response*						
Δ height, SDS^b^	129	0.99	0.57	1.38	1.00	0.52
Δ HV, cm/year	107	4.6	3.1	7.0	5.1	3.3
HV, cm/year	129	10.4	8.2	12.0	10.2	2.5
HV for age and sex, SDS	115	1.51	0.09	3.50	1.91	2.23
*Responsiveness*						
HV for first-year GH treatment^c^, SDS	129	0.26	−0.31	0.91	0.31	0.88
Index of responsiveness (with GH peak)	123	0.02	−0.59	0.71	0.07	1.13
Index of responsiveness (without GH peak)	123	0.13	−0.51	0.90	0.21	1.13
*Prediction of nFAH*						
Predicted nFAH (with GH peak)^d^	123	−0.87	−1.37	−0.36	−0.84	0.87
Predicted nFAH (without GH peak)^d^	123	−0.85	−1.41	−0.37	−0.85	0.87
**At nFAH**						
Age, years (boys)	83	18.3	17.6	19.2	18.9	2.3
Age, years (girls)	46	16.3	15.5	17.7	16.7	1.7
Age stop GH treatment, years (boys)	83	16.8	16.1	17.6	16.9	1.3
Age stop GH treatment, years (girls)	46	15.3	14.7	16.4	15.6	1.3
Growth since stop GH treatment, cm	129	0.6	0.0	1.1	1.3	2.4
Duration GH therapy, years	129	9.9	7.5	11.7	9.7	2.6
Duration GH therapy before pubertal onset, years	122	5.5	3.2	7.8	5.6	2.7
nFAH, SDS (A21)	129	−1.45	−2.02	−0.67	−1.40	1.10
nFAH, SDS (CA)	129	−1.19	−1.91	−0.41	−1.17	1.08
nFAH minus MPH, SDS (A21)	124	−0.38	−0.96	0.23	−0.39	0.94
nFAH minus MPH, SDS (CA)	124	−0.17	−0.70	0.45	−0.16	0.94
Total Δ height, SDS^e^ (A21)	129	1.84	1.19	2.69	1.99	1.13
Total Δ height, SDS^e^(CA)	129	2.05	1.55	2.97	2.23	1.09
BMI, SDS (A21)	110	−0.55	−1.47	0.29	−0.49	1.36
BMI, SDS (CA)	110	−0.15	−1.05	0.61	−0.19	1.27

GH, growth hormone; nFAH, near final adult height; SDS, standard deviation score; MPH, midparental height; BMI, body mass index; HV, height velocity; cm, centimeter; A21, SDS calculated at age 21 years; CA, SDS calculated at chronological age;

a change in BMI SDS after first-year GH treatment;

b gain in height SDS after first-year GH treatment;

c growth targets for first-year GH response by Ranke et al.;

d prediction model for nFAH by Ranke et al.;

e *gain in height SDS from start of GH treatment until nFAH*.

### First-Year Response and Responsiveness to GH Treatment (Table 1)

After the first year of GH therapy, the median ΔHt SDS was 0.99, the mean (± SD) first-year HV was 10.2 cm/year (±2.5) or 1.91 SD (±2.23), and the mean ΔHV was 5.1 cm/year (±3.3). The mean HV SDS on the first-year GH treatment response curve by Ranke et al. was 0.31 (±0.88). The mean IoR was respectively 0.07 (±1.13) and 0.21 (±1.13), for the formula with and without max. GH peak. The mean predicted nFAH SDS was −0.84 (±0.87) with, and −0.85 (±0.87) without the maximum GH peak included.

### Final Height Outcome After GH Treatment

Near FAH after GH treatment is listed in Table 1. The mean duration of GH therapy was 9.7 years, with a mean duration before pubertal onset of 5.6 years. nFAH was attained at a mean age of 16.7 years in girls and 18.9 years in boys. For girls, mean nFAH was 157.6 cm ± 7.0 (−1.52 SD ± 1.19, and −1.34 ± 1.15, resp. for A21 and CA). For boys, mean nFAH was 172.1 cm ± 7.1 (−1.33 SD ± 1.06, and −1.08 SD ± 1.04, resp. for A21 and CA). Twenty six and 22% of patients had a nFAH < −2.0 SD, resp. for A21 and CA. Mean nFAH SDS minus MPH SDS was −0.39 (A21) and −0.16 (CA). Twelve and 10% of patients had a nFAH SDS minus MPH SDS <−1.3, resp. for A21 and CA. The median total increase in height SDS was 1.99 (A21) and 2.23 (CA). Median total ΔHt SDS was comparable in girls and boys [mean difference 0.13 SD (A21) and 0.22 SD (CA); *p* = 0.5]. Sixteen and 12% of patients had a total ΔHt SDS < 1, resp. for A21 and CA.

### Logistic Regression Analysis

**Figure 1:**
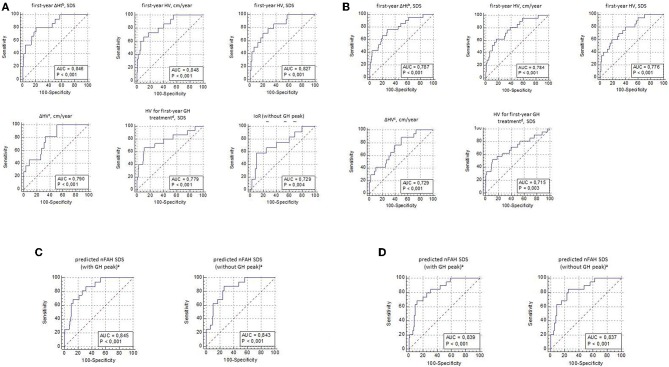
**(A)** ROC curve analysis for first-year response and responsiveness parameters, with its sensitivity and specificity to predict total ΔHt SDS^a^ <1(CA). CA, SDS calculated at chronological age; SDS, standard deviation score; cm, centimeter; HV, height velocity; GH, growth hormone; IoR, index of responsiveness; AUC, area under the ROC curve; ^a^gain in height SDS f rom start of GH treatment until near final adult height; ^b^gain in height SOS after first-year GH treatment; ^c^HV during first-year GH treatment minus HV during pretreatment year; ^d^growth targets for first-year GH response by Ranke et al. **(B)** ROC curve analysis for first-year response and responsiveness parameters, with its sensitivity and specificity to predict total ΔHt SDS^a^ <1(A21). A21, SDS calculated at age 21years; SDS, standard deviation score; cm, centimeter; HV, height velocity; GH, growth hormone; AUC, area under the ROC curve; ^a^gain in height SDS f rom start of GH treatment until near final adult height; ^b^gain in height SDS after first-year GH treatment; ^c^HV during first-year GH treatment minus HV during pretreatment year; ^d^growth targets for first-year GH response by Ranke et al. **(C)** ROC curve analysis for predicted nFAH after first-year GH treatment^a^, with its sensitivity and specificity to predict nFAH SDS <−2 (Prader, CA). nFAH, near final adult height; GH, growth hormone; SDS, standard deviation score; CA, SDS calculated at chronological age; AUC, area under the ROC-curve; ^a^prediction model f or nFAH after first-year GH treatment by Ranke et al. **(D)** ROC curve analysis for predicted nFAH after first-year GH treatment^a^, with its sensitivity and specificity to predict nFAH SDS <−2 (Prader, A21). nFAH, near final adult height; GH, growth hormone; SDS, standard deviation score; A21, SDS calculated at age 21 years; AUC, area under the ROC-curve; ^a^prediction model f or nFAH after first-year GH treatment by Ranke et al.

ROC curve analysis was performed for all first-year response and responsiveness parameters [ΔHt SDS, HV for age and sex (cm/year and SDS), ΔHV (cm/year), HV SDS for first-year GH treatment, IoR, predicted nFAH SDS] in relation to the studied poor final outcome parameters (total ΔHt SDS <1, nFAH SDS < −2, and nFAH SDS—MPH SDS < −1.3) (Figures 1A–D). Only ROC-curves with an AUC ≥70% were further analyzed.

Tables 2A–D show the thresholds with their sensitivity and specificity of the different tests vs. the different outcomes. The thresholds for the tests currently proposed in the literature are set in bold.

**Table 2A:** ROC curve analysis: cut-off values for first-year response and responsiveness parameters, with its sensitivity and specificity to predict total ΔHt SDS < 1^a^ (CA).

**ΔHt^**b**^, SDS**	**Sensitivity (%)**	**Specificity (%)**	**HV, cm/year**	**Sensitivity (%)**	**Specificity (%)**	**HV for age and sex, SDS**	**Sensitivity (%)**	**Specificity (%)**
0.20	20	100	5.9	13	100	−1.93	14	100
0.28	33	98	6.5	33	98	−1.00	29	97
*0.35*	*40*	*95*	6.6	40	97	–*0.85*	*43*	*95*
**0.50**	**60**	**86**	*6.8*	*47*	*95*	−0.38	57	88
0.57	73	82	7.4	60	90	0.09	71	80
0.61	80	79	8.0	67	85	**1.00**	**78**	**67**
1.00	87	54	8.9	80	68	1.22	86	63
1.03	93	50	10.8	93	49	2.48	93	45
1.14	100	43	11.0	100	45	2.56	100	43
AUC: 85% (95% CI: 77–90%)			AUC: 85% (95% CI: 77–91%)			AUC: 83% (95% CI: 75–89%)		
**ΔHV**^**c**^**, cm/year**	**Sensitivity (%)**	**Specificity (%)**	**HV for first-year GH treatment**^**d**^**, SDS**	**Sensitivity (%)**	**Specificity (%)**	**IoR (without GH peak)**	**Sensitivity (%)**	**Specificity (%)**
−2.3	27	100	−1.57	13	100	−2.24	0	100
1.2	36	97	−1.14	20	98	−1.82	8	97
*1.3*	*36*	*95*	–**1.00**	**33**	**97**	–*1.57*	*17*	*95*
1.6	45	92	–*0.83*	*40*	*95*	–**1.28**	**17**	**92**
**3.2**	**45**	**74**	−0.68	53	90	−0.97	58	90
3.8	72	69	−0.19	73	73	−0.40	67	75
4.9	82	49	0.43	87	46	0.43	83	40
5.1	100	49	1.03	93	24	0.69	92	32
			1.46	100	12	1.16	100	21
AUC: 79% (95% CI: 70– 86%)			AUC: 78% (95% CI: 70–85%)			AUC: 73% (95% CI: 64–81%)		

*CA, SDS calculated at chronological age; SDS, standard deviation score; cm, centimeter; HV, height velocity; GH, growth hormone; IoR, index of responsiveness; AUC, area under the ROC curve; CI, confidence interval*,

a gain in height SDS from start of GH treatment until near final adult height;

b gain in height SDS after first-year GH treatment;

c HV during first-year GH treatment minus HV during pretreatment year;

d *growth targets for first-year GH response by Ranke et al. bold, currently used FYGR criteria; italic, FYGR criteria at 95% specificity*.

**Table 2B:** ROC curve analysis: cut-off values for first-year response and responsiveness parameters, with its sensitivity and specificity to predict total ΔHt SDS < 1^a^ (A21).

**ΔHt, SDS^**b**^**	**Sensitivity (%)**	**Specificity (%)**	**HV, cm/year**	**Sensitivity (%)**	**Specificity (%)**	**HV for age and sex, SDS**	**Sensitivity (%)**	**Specificity (%)**
0.20	14	100	5.9	10	100	−1.93	10	100
0.30	29	98	6.6	29	98	−0.94	35	98
*0.37*	*43*	*95*	*6.7*	*33*	*95*	–*0.60*	*40*	*95*
**0.50**	**48**	**86**	7.6	52	90	−0.22	50	85
0.60	62	80	8.3	62	80	0.11	60	80
0.69	71	73	8.9	67	70	0.60	65	73
0.99	81	56	9.9	81	61	**1.00**	**70**	**68**
1.03	86	51	10.8	86	50	1.48	80	60
1.14	90	44	11.0	90	45	2.56	90	43
1.22	95	39	11.1	95	42	2.63	95	40
1.56	100	16	12.9	100	17	3.50	100	29
AUC: 79% (95% CI: 71–85%)			AUC: 78% (95% CI: 70–85%)			AUC: 78% (95% CI: 69–85%)		
**ΔHV**^**c**^**, cm/year**	**Sensitivity (%)**	**Specificity (%)**	**HV for first-year GH treatment**^**d**^**, SDS**	**Sensitivity (%)**	**Specificity (%)**			
−2.4	18	100	−1.57	10	100			
*1.4*	*29*	*95*	–**1.00**	**29**	**98**			
1.8	35	90	–*0.83*	*33*	*95*			
**3.0**	**41**	**79**	−0.59	52	89			
3.4	47	74	−0.32	57	81			
3.9	65	67	−0.19	62	73			
4.4	71	61	0.14	71	60			
5.1	82	50	0.43	81	46			
6.0	88	40	1.03	90	24			
6.9	94	30	1.46	95	12			
7.3	100	27	1.85	100	4			
AUC: 73% (95% CI: 63–81%)			AUC: 72% (95% CI: 63–79%)					

*A21, SDS calculated at age 21 years; SDS, standard deviation score; HV, height velocity; cm, centimeter; GH, growth hormone; AUC, area under the ROC curve; CI, confidence interval*,

a gain in height SDS from start of GH treatment until near final adult height;

b gain in height SDS after first-year GH treatment;

c HV during first-year GH treatment minus HV during pretreatment year;

d *growth targets for first-year GH response by Ranke et al. bold, currently used FYGR criteria; italic, FYGR criteria at 95% specificity*.

**Table 2C:** ROC curve analysis: cut-off values for predicted nFAH after first-year GH treatment^a^, with its sensitivity and specificity to predict nFAH SDS <−2 (Prader, CA).

**Predicted nFAH SDS (with GH peak)^**a**^**	**Sensitivity (%)**	**Specificity (%)**	**Predicted nFAH SDS (without GH peak)^**a**^**	**Sensitivity (%)**	**Specificity (%)**
−2.62	19	100	−2.53	25	100
*−1.94*	*25*	*95*	–*2.02*	*25*	*95*
−1.74	44	91	−1.77	44	91
−1.65	63	90	−1.70	63	90
−1.28	75	79	−1.51	69	83
−1.17	81	74	−1.21	81	76
−1.04	88	68	−1.20	88	74
−0.87	94	55	−0.78	94	52
−0.69	100	47	−0.64	100	44
AUC: 85% (95% CI: 77–90%)			AUC: 84% (95% CI: 77–90%)		

nFAH, near final adult height; GH, growth hormone; SDS, standard deviation score; CA, SDS calculated at chronological age; AUC, area under the ROC-curve; CI, confidence interval;

a *prediction model for nFAH after first-year GH treatment by Ranke et al. italic, FYGR criteria at 95% specificity*.

**Table 2D:** ROC curve analysis: cut-off values for predicted nFAH after first-year GH treatment^a^, with its sensitivity and specificity to predict nFAH SDS <−2 (Prader, A20).

**Predicted nFAH SDS (with GH peak)^**a**^**	**Sensitivity (%)**	**Specificity (%)**	**Predicted nFAH SDS (without GH peak)^**a**^**	**Sensitivity (%)**	**Specificity (%)**
−2.62	16	100	−2.53	21	100
–*1.95*	*21*	*95*	–*1.91*	*26*	*95*
−1.67	63	91	−1.70	63	91
−1.50	68	88	−1.51	68	85
−1.28	74	80	−1.25	74	77
−1.04	84	69	−1.20	84	75
−0.87	89	56	−0.78	89	53
−0.69	95	47	−0.64	95	44
−0.61	100	41	−0.48	100	38
AUC: 84% (95% CI: 76–90%)			AUC: 84% (95% CI: 76–90%)		

*nFAH, near final adult height; GH, growth hormone; SDS, standard deviation score; A21, SDS calculated at age 21 years; AUC, area under the ROC-curve; CI, confidence interval*,

a *prediction model for nFAH after first-year GH treatment by Ranke et al. italic, FYGR criteria at 95% specificity*.

Tables 2A,B show cut-off values for first-year response and responsiveness parameters, with its sensitivity and specificity to predict total ΔHt SDS <1 (CA and A21). The first-year response criterion ΔHt SDS <0.5 had a relatively low specificity (86%) to predict a total ΔHt SDS <1. The corresponding sensitivity was 60%. The other proposed first-year response and responsiveness criteria had a specificity of 67–97%, with corresponding sensitivities of 17–78%.

To predict a total ΔHt SDS <1 (CA) with a 95% specificity (in italic) the following threshold levels were found: ΔHt <0.35 SD; HV <6.8 cm/year; HV < −0.85 SD for age and sex; ΔHV < 1.3 cm/year; HV < −0.83 SD for first-year GH treatment by Ranke et al.; IoR (without GH peak) < −1.57. The corresponding sensitivities were respectively 40, 47, 43, 36, 40, and 17%. The total ΔHt SDS of the good final responders who were wrongly diagnosed as poor final responders (according to the above criteria) varied between 1.08 and 2.57.

Tables 2C,D show cut-off values for predicted nFAH after first-year GH treatment, with its sensitivity and specificity to predict nFAH SDS <−2.0 (Prader, CA and A20). A predicted nFAH after first-year GH treatment < −1.94 SD (model with GH peak) and < −2.02 (model without GH peak) predicted nFAH SDS <−2 (CA) with 95% specificity and 25% sensitivity. The nFAH SDS of the good final responders who were wrongly diagnosed as poor final responders (according to the above criteria) varied between −1.98 and −1.28.

For all FYGR parameters in relation to nFAH minus MPH SDS < −1.3, the AUC's were <70% and therefore not further analyzed.

### Comparison of the Good and the Poor Final Height Responders

The patients having a total ΔHt SDS in the highest quartile had a significantly lower height SDS at start of GH treatment compared with the patients in the lowest ΔHt SDS quartile (−3.78 SD vs. −3.03 SD; *p* < 0.001) (Table 3). They also had a significantly higher first-year ΔHt SDS (1.50 SD vs. 0.61 SD; *p* < 0.001). Therefore, they reached a comparable height SDS after the first year of GH treatment (−2.28 SD vs. −2.41 SD; *p* = 0.5). The total ΔHt was 3.71 SD for the good (highest quartile) and 0.98 SD for the poor (lowest quartile) total ΔHt responders. The poor total ΔHt SDS responders had a significantly lower birth weight, shorter parents, and a less severe GHD. They started GH at an older age, with a taller height, and lower BMI, and received GH for a shorter period than the good total ΔHt SDS responders.

**Table 3:** Comparison of poor and good final responders.

	**25% poorest total ΔHt SDS^§^**			**25% best total ΔHt SDS^§^**			***p*-value**	**25% poorest nFAH SDS^§^^&^**			**25% best nFAH SDS^§^^&^**			***p*-value**
**Background**	**n**	**Mean**	**SD**	**n**	**Mean**	**SD**		**n**	**Mean**	**SD**	**n**	**Mean**	**SD**	
Birth weight, SDS	28	−0.53	0.73	30	−0.05	0.89	<0.05							
Father height, SDS	29	−1.43	1.04	30	−0.41	1.19	<0.01	29	−1.66	1.21	30	0.35	1.01	<0.001
Mother height, SDS	29	−1.42	0.99	30	−0.54	1.05	<0.01	29	−1.67	1.04	30	0.17	0.90	<0.001
MPH, SDS	29	−1.43	0.69	30	−0.50	0.92	<0.001	29	−1.66	0.91	30	0.24	0.80	<0.001
Maximum GH peak, μg/L	32	6.4	2.4	32	2.7	1.6	<0.001	32	4.8	2.9	32	3.0	1.9	<0.01
**At start GH treatment**														
Age, years	32	7.2	2.4	32	5.8	2.3	<0.05							
Height, SDS	32	−3.03	0.71	32	−3.78	0.8	<0.001	32	−3.88	0.89	32	−3.10	0.91	<0.01
Height minus MPH, SDS	29	−1.54	0.83	30	−3.25	0.97	<0.001	29	−2.25	1.33	30	−3.19	0.81	<0.01
BMI, SDS	32	−0.69	0.98	32	−0.07	1.08	<0.05							
HV during pretreatment year, cm/year	*27*	*5.7*	*2.1*	*25*	*4.8*	*2.2*	*0.2*	*27*	*5.2*	*2.1*	*28*	*4.8*	*2.2*	*0.5*
**After first-year GH treatment**														
Height, SDS	*32*	–*2.41*	*0.63*	*32*	–*2.28*	*0.83*	*0.5*	32	−2.98	0.80	32	−1.65	0.72	<0.001
Height minus MPH, SDS	29	−0.91	0.79	30	−1.76	1.06	<0.01							
*Growth response*														
Δ height, SDS^b^	32	0.61	0.36	32	1.50	0.44	<0.001	32	0.90	0.47	32	1.45	0.47	<0.001
Δ HV, cm/year	27	2.9	3.0	25	7.8	3.1	<0.001	27	4.6	3.4	25	7.8	3.3	<0.01
HV, cm/year	32	8.3	1.8	32	12.6	1.9	<0.001	32	9.4	2.3	32	12.7	2.0	<0.001
HV for age and sex, SDS	31	0.35	1.60	26	4.20	1.73	<0.001	31	1.24	2.03	26	4.36	1.96	<0.001
*Responsiveness*														
HV for first-year GH treatment^c^, SDS	32	−0.16	0.87	32	0.89	0.80	<0.001	32	0.02	0.94	32	1.01	0.83	<0.001
Studentized residual (with GH peak)	29	−0.24	1.07	30	0.73	1.24	<0.01	29	−0.11	1.16	30	0.76	1.26	<0.01
Studentized residual (without GH peak)	29	−0.34	0.93	30	0.99	1.08	<0.001	29	−0.01	1.09	30	1.00	1.19	<0.01
*Prediction of nFAH*														
Predicted nFAH (with GH peak)^d^	29	−1.20	0.57	30	−0.37	0.77	<0.001	29	−1.56	0.77	30	0.36	0.64	<0.001
Predicted nFAH (without GH peak)^d^	29	−1.21	0.57	30	−0.39	0.79	<0.001	29	−1.57	0.79	30	0.35	0.66	<0.001
**At puberty onset**														
Duration GH therapy before puberty, years	31	4.9	2.5	32	6.7	2.5	<0.01							
Height, SDS	31	−2.10	0.67	31	−0.84	1.00	<0.001	31	−2.40	0.86	31	−0.31	0.85	<0.001
Δ height, SDS	31	0.94	0.46	31	2.91	0.96	<0.001	31	1.48	0.79	31	2.67	1.18	<0.001
**At nFAH**														
Duration GH therapy, years	32	8.9	2.5	32	11.0	2.2	<0.01							
nFAH, SDS^*^	32	−2.31	0.71	32	−0.30	0.95	<0.001	32	−2.34^&^	0.49	32	0.90^&^	0.49	<0.001
nFAH, SDS^§^	32	−2.05	0.71	32	−0.11	0.88	<0.001	32	−2.26^&^	0.50	32	0.99^&^	0.47	<0.001
nFAH minus MPH, SDS^*^	29	−0.78	0.67	30	0.17	0.99	<0.001	29	−1.04^&^	0.93	28	0.31^&^	0.95	<0.001
nFAH minus MPH, SDS^§^	29	−0.53	0.73	30	0.41	0.96	<0.001	29	−0.96^&^	0.91	28	0.40^&^	0.92	<0.001
Total Δ height^e^, SDS^*^	32	0.71	0.47	32	3.48	0.85	<0.001	32	1.76^&^	0.87	32	4.12^&^	1.16	<0.001
total Δ height^e^, SDS^§^	32	0.98	0.42	32	3.71	0.71	<0.001	32	1.84^&^	0.84	32	4.20^&^	1.14	<0.001
BMI, SDS^*^	25	−0.99	1.16	28	−0.05	1.17	<0.01							
BMI, SDS^§^	25	−0.63	1.03	28	0.21	1.14	<0.01							

Characteristics: background, at GH start, after first year, at puberty onset, at nFAH GH, growth hormone; nFAH, near final adult height; SDS, standard deviation score; MPH, midparental height; BMI, body mass index; HV, height velocity; SDS, standard deviation score; cm, centimeter;

* SDS calculated at 21 years;

§ SDS calculated at chronological age;

& SDS calculated with Prader references;

a change in BMI SDS after first-year GH treatment;

b gain in height SDS after first-year GH treatment;

c growth targets for first-year GH response by Ranke et al.;

d prediction model for nFAH by Ranke et al.;

e *gain in height SDS from start of GH treatment until nFAH*.

The patients in the highest quartile nFAH SDS had a significantly higher height SDS at start compared to the patients in the lowest quartile nFAH SDS (−3.10 SD vs. −3.88 SD; *p* < 0.01) (Table 3). Delta height SDS after the first year, at onset of puberty and at nFAH was significantly higher in the good responders. They had also taller parents and more severe GHD.

## Discussion

In this study of a cohort of GHD patients treated with GH extracted from the Belgian Registry we found that the mean nFAH was still below average and 10–22% of the patients had a poor final height outcome. ROC-analysis showed that the currently used FYGR criteria had low specificities and sensitivities to detect PFHO.

Our final height outcome data in Belgian patients are comparable with the results of a Swedish (2) and Canadian (4) study, using the same criteria for nFAH, where idiopathic GHD children were treated with a similar GH dose for a mean period of 8.6 and 5.4 years, respectively: up to 84 and 90% obtained a nFAH SDS > −2. We previously reported in a smaller group of Belgian idiopathic GHD patients a comparable nFAH (170.4 cm in males and 158 cm in females after a mean treatment duration of 5.2 years) and a similar response rate (84% had a nFAH within normal limits) (22).

Near FAH was taken as a proxy of FAH as an outcome parameter, as many patients usually stop GH treatment and disappear from follow-up when growth slows down to less than 2 cm per year and before adult height is reached (23). To overcome this problem, nFAH SDS could be calculated at a reference age of 21 years instead of the actual chronological age. This underestimates the real Ht SDS since most adolescents will still gain a few centimeters. On the other hand, since the mean height of the reference population also increases between 16 and 21 years, nFAH SDS at the actual chronological age will overestimate the real Ht SDS. We therefore calculated nFAH SDS both with age set at 21 years (worst case scenario) and at chronological age (best case scenario), accepting that the first method will underestimate and the second will overestimate the actual FAH SDS.

This ROC-analysis showed that the classically proposed threshold levels for first-year growth response and responsiveness parameters had a low sensitivity and specificity to predict a poor near final height outcome. For example, first-year ΔHt SDS <0.5 had a sensitivity of 60%. This means that 60% of the poor final responders (total ΔHt SDS < 1.0) had a poor first-year response (first-year ΔHt SDS < 0.5), and 40% (100-sensitivity) of the poor final responders had a good first-year response (first-year ΔHt SDS > 0.5). The corresponding specificity was 86%, meaning 86% of the good final responders had a good first-year response, and 14% (100-specificity) of the good final responders had a poor first-year response. Thus, first-year ΔHt SDS < 0.5 correctly identified 60% of the poor final responders, but misdiagnosed 14% of the good final responders as poor responders. In order to misdiagnose good final responders as few as possible (5%), we decided to set the specificity of the FYGR parameters at 95% and determined the test cut-off values. At these newly defined threshold values, the sensitivity to detect poor final height responders decreased considerably. Of course, every physician can chose the specificity required by the local circumstances. The FYGR threshold values that best predicted total ΔHt SDS < 1 with a 95% specificity were: Δ Ht SDS < 0.35; HV SDS < −0.85, HV for first-year GH treatment SDS < 0.83, and Δ HV < 1.3 cm/year. On the other hand, predicted nFAH SDS (with GH peak) < −1.94, and predicted nFAH SDS (without GH peak) < −2.02 performed best to detect nFAH < −2 SD (Prader) with a 95% specificity. These criteria only correctly identify 25–43% (= sensitivity) of the patients with a poor final outcome (= 3.8–5.2% of the total population). At a specificity of 95%, 5% of good final responders is wrongly diagnosed as poor final responder (= 4.2–4.4% of the total population). At these cut-offs the amount of correctly diagnosed poor responders equals the amount of false positives due to the relatively low prevalence of poor responders.

Several parameters, such as birth weight, midparental height, age at start, max. GH peak in provocation test, height at start, and IoR after the first year of GH treatment were found to differ between patients with a good or a poor final height outcome. Not surprisingly, these parameters are also used in prediction models for nFAH, such as in the model by Ranke et al. (16). However, these parameters were found to only explain 60% of the variability. An incorrect diagnosis of GHD or the presence of another growth limiting condition at start of GH as well as several conditions during the GH course, such as GH dose adaptations during the first year, poor compliance after the first year of GH treatment as well as variability in pubertal onset, pubertal growth and bone age progression may all explain the poor predictability of the FAH outcome in GH treated children.

This is the first study evaluating the final height predictability of the currently used first year growth response parameters, putting them in a new long-term perspective. However, this study has also several shortcomings. Treatment adherence and the persistence of the GHD were not assessed routinely in the studied cohort. Secondly, the size of the cohort was rather small, despite the national recruitment of patients.

Despite FYGR criteria were found not to be suitable for detecting poor or good final responders without too many misdiagnoses, it is still important to evaluate first-year response to GH to identify poor compliance, improper administration of GH, additional health problems, poor nutrition, impaired GH sensitivity due to mutations in the GH-IGF-1 axis genes, incorrect initial diagnosis, etc.

In conclusion, the currently used first-year growth response and responsiveness parameters perform poorly as predictors of a poor final height outcome after long-term GH treatment in prepubertal GHD children, due to low sensitivities and/or specificities and the low prevalence of poor responders in this group. The FYGR parameters may perform better in indications with more poor responders or when more stringent criteria for poor near final height outcome (e.g., ΔHt SDS >1.5) are used.

## Flow Chart Patient Selection

**Table:** 

*n = 1230, “Idiopathic GHD” (=GHD not secondary to a treatment of a tumor or not NSD) with or without syndromes*	
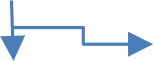	*Idiopathic GHD with syndromes or dysmorphic features (*n* = 187)*
*n = 1043, Idiopathic GHD, Idiop congenital, idiopathic SOD, Idiopathic genetic, Idiopathic infection, Idiopathic midline defect*	
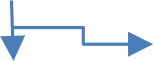	*Start GH before 1986 (n = 45)*
*n = 998*	
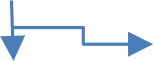	*SGA (n = 132)*
*n = 866*	
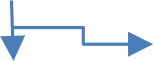	*Age start GH < 2 year (n = 81)*
*n = 785*	
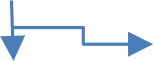	*Age at start GH treatment >11 year (females) (n = 94); >12 year (males) (n = 137)*
*n = 554*	
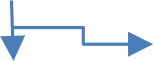	*Duration of GH <4 year or interruption >6 months (n = 40)*
*n = 514*	
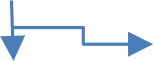	*Age at nFAH ≤ 15 year (females) and ≤17 year (males) or GH treatment not stopped (n = 242)*
*n = 272*	
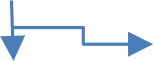	*GH peak in provocation test >10 ng/ml or only 1 provocation test performed (n = 14)*
*n = 258*	
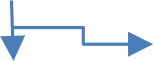	*Too many missing visits (n = 2)*
*n = 256*	
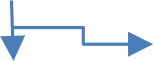	*Not prepubertal after 1 year GH therapy (n = 13) or pubertal stage unknown (n = 8)*
*n = 235*	
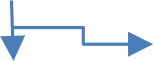	*GH treatment not daily or not recombinant human GH (n = 31)*
*n = 204*	
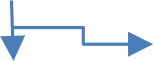	*No adult height (defined as height velocity < 2cm/year) (n = 75)*
*n = 129*	

## Data Availability Statement

The datasets generated for this study are available on request to the corresponding author.

## Ethics Statement

The studies involving human participants were reviewed and approved by Ethical Commitee of the University of Brussels and University Hospital of Brussels. Written informed consent to participate in this study was provided by the participants' legal guardian/next of kin.

## Author Contributions

SS, JD, MT, ST, VB, and RR contributed to the conception, design of the study and wrote sections of the manuscript. SS and MT organized the database. SS performed the statistical analysis and wrote the first draft of the manuscript. All authors contributed to manuscript revision, read, and approved the submitted version.

### Conflict of Interest

RR is the founder of PendoCon, has received consulting fees from Pfizer and Ferring, and is a member of the Pfizer iGRO Advisory Board. The remaining authors declare that the research was conducted in the absence of any commercial or financial relationships that could be construed as a potential conflict of interest.
